# Innovations in functional genomics and molecular breeding of pea: exploring advances and opportunities

**DOI:** 10.1007/s42994-023-00129-1

**Published:** 2024-01-30

**Authors:** Baizhi Chen, Yan Shi, Yuchen Sun, Lu Lu, Luyao Wang, Zijian Liu, Shifeng Cheng

**Affiliations:** Agricultural Genomics Institute at Shenzhen (AGIS), Chinese Academy of Agricultural Sciences (CAAS), Shenzhen, China

**Keywords:** Pea, Genome study, Agronomic traits, Breeding

## Abstract

The garden pea (*Pisum sativum* L.) is a significant cool-season legume, serving as crucial food sources, animal feed, and industrial raw materials. The advancement of functional genomics over the past two decades has provided substantial theoretical foundations and progress to pea breeding. Notably, the release of the pea reference genome has enhanced our understanding of plant architecture, symbiotic nitrogen fixation (SNF), flowering time, floral organ development, seed development, and stress resistance. However, a considerable gap remains between pea functional genomics and molecular breeding. This review summarizes the current advancements in pea functional genomics and breeding while highlighting the future challenges in pea molecular breeding.

## Introduction

The garden pea (*Pisum sativum* L., 2*n* = 14) is a cold-season, annual climbing legume, ranking as one of the eight foundational crops and originally domesticated in the Near East and the Mediterranean Basin (Singh et al. 2019). Noted for its rich content of protein, fiber, vitamins, and minerals, peas are acclaimed for their exceptional nutritional composition (Singh et al. 2019; Paul and Southgate 1978). Beyond human consumption, peas have been utilized in animal feed, green manure, and various industrial applications (Piotrowska-Długosz and Wilczewski 2020; Bastianelli et al. 1998). Ranked as the fourth-largest leguminous crop after soybeans (*Glycine max*), peanuts (*Arachis hypogaea*), and common beans (*Phaseolus vulgaris*), the planting area for dry peas reached 7.04 million hectares (Mha), and for fresh peas, 2.59 Mha in 2021. However, with a yield of only 1700 kg/ha, peas lag significantly behind other leguminous crops (http://www.fao.org/faostat/). Given the increase in world population and reduction in arable land, enhancing pea yield has become a crucial goal in breeding. In addition, the climbing nature of peas necessitates manual trellising, resulting in higher labor costs. Therefore, current breeding goals include not only increasing yield but also modifying the plant structure to simplify cultivation. Over the past decade, the development of functional genomics in peas, especially with the public release of the pea reference genome and the integration of multi-omics technologies, has deepened our understanding of the growth and developmental processes in peas. Here, we rereview the evolution of pea functional genomics, with a focus on loci and genes favorable for breeding, and discuss the future genes and challenges in molecular breeding of peas.

## The progress of pea genome studies

With the advent of the genomics era in plant science, the pea plant, possessing a sizeable genome of 4.45 GB, has trailed significantly in genomic research compared to other leguminous plants (Doležel and Greilhuber 2010; Smýkal et al. 2012). As reference genomes for legumes such as *Lotus japonicus* (Sato et al. 2008), soybean (Schmutz et al. 2010), and *Medicago truncatula* (Young et al. 2011) became available, the pea has gradually lost its stature as a premier model organism in the legume family. This extensive genome of the pea is attributed to its content, which consists of 75–95% repetitive sequences (Flavell et al. 1974; Murray et al. 1981). More recent studies have confirmed that these sequences, representing about 76% of pea nuclear DNA, belong to highly diverse families of sequences with high to moderate repetition (Macas et al. 2015). These intricate repetitive sequences undeniably posed significant challenges to the early genome assembly reliant on Next-Generation Sequencing (NGS) technology.

Although the limitations of second-generation sequencing make it challenging to assemble the entire genome of the pea plant, the advent of transcriptome sequencing has enabled researchers to attempt de novo assembly at the transcript level of pea genes (Table 1) (Sudheesh et al. 2015; Alves‐Carvalho et al. 2015). Alves‐Carvalho et al. utilized 20 cDNA libraries from ‘Caméor’, comprising a variety of subterranean and aerial plant tissues, diverse developmental stages, and nutritional conditions, to generate a comprehensive set of Unigene expressed sequences (Alves‐Carvalho et al. 2015). Concurrently, Sudheesh et al. leveraged two commonly cultivated Australian field pea cultivars, ‘Kaspa’ and ‘Parafield’, to generate a comprehensive assembled and annotated transcriptome set for field pea (Sudheesh et al. 2015). With the rise of third-generation sequencing and the gradual reduction in the cost of second-generation sequencing, the first chromosome-level reference genome of pea was published in 2019 (Kreplak et al. 2019). Subsequently, for the study of the yellow pod trait in Mendel’s seven traits, Shirasawa et al. assembled the reference genome of the yellow pod material JI128 (Shirasawa et al. 2021). Following this, with the advancement of Hi-C technology, Yang et al. used a combination of third-generation sequencing and Hi-C mounting to assemble the reference genome of China’s main cultivated variety ‘ZW6’ (Yang et al. 2022a). This resulted in a significant improvement in both completeness and accuracy compared to previous reference genomes.

**Table 1:** Information of pea reference genomes

Accession information	Method	Accession number	References
Caméor (cultivars)	De novo assembly of RNA-seq data	PRJNA267198	Sudheesh et al. (2015)
Kaspa (cultivars)	De novo assembly of RNA-seq data	PRJNA277074	Alves-Carvalho et al. (2015)
Parafield (cultivars)		PRJNA277076	
Caméor (cultivars)	De novo sequencing and assembly (ONT + NGS)	PRJEB31320	Kreplak et al. (2019)
JI128 (genetic stock)	De novo sequencing and assembly (PacBio RSII + NGS)	PRJDB10540	Shirasawa et al. (2021)
ZW6 (cultivars)	De novo sequencing and assembly (HiFi + Hi-C)	PRJNA730094	Yang et al. (2022a)
118 cultivars and wilds	Re-sequencing	PRJNA730094	Yang et al. (2022a)

## Germplasm resources and databases

Peas possess a rich germplasm resource characterized by a vast array of variations. The Plant Germplasm Introduction and Testing Research Station in the United States has amassed 5400 pea germplasm resources, complete with phenotypic and genotypic data. Similarly, the Australian Temperate Field Crop Collection in Australia has gathered 6567 accessions, and the John Innes Centre in the United Kingdom has collected 3557 accessions, both replete with phenotypic and genotypic data (Smýkal et al. 2012). In addition, the Institute of Crop Sciences, CAAS, in China has acquired 3837 pea germplasm resources. Studies on the structure and genetic diversity of core germplasm populations have illuminated the process of pea domestication (Yang et al. 2022a; Weeden 2018).

Peas, being difficult to genetically transform, necessitate the use of mutant populations to facilitate gene cloning, functional analysis, and mutation breeding. Extensive exploration of different mutagenic conditions has led to the development of various mutation methods, resulting in the creation of numerous mutants. Principal collections of pea mutants encompass. The primary collections of pea mutants include: (1) The John Innes Collection in Norwich, UK, with 575 accessions; (2) The IPGR collection in Plovdiv, Bulgaria, with 122 accessions; (3) A population with TILLING-induced localized lesions, consisting of 4817 lines; and (4) A set of 93 symbiotic mutants (Sagan and Duc 1996; Sagan et al. 1994). Researchers have conducted comprehensive investigations into various traits of peas by utilizing mutants derived from different mutagenic conditions. examples include the cloning of the *Tendril-less* (*Tl*) locus from fast neutron mutants (Hofer et al. 2009), the cloning of the *Elephant-ear-like leaf1* (*ELE1*) locus from ethylmethane sulphonate (EMS) mutants (Li et al. 2019), and the cloning of the *Keeled Wings* (*K*) locus from x-ray mutagenesis (Wang et al. 2008). In addition, the Mendelian flower color gene was successfully cloned using mutants (Hellens et al. 2010). Moreover, pea mutation breeding, initiated in the early 1940s, has proven highly successful. A prime example of this is the development of a semi-leafless pea variety, named ‘Wasata’ by Poland in 1979, utilizing gamma-ray mutagenesis. This advancement markedly increased the pea’s resistance to lodging, without compromising its yield (Solanki et al. 2011).

Several databases pertinent to pea genomics, genetic markers, and germplasm have been established. Notably, these freely accessible databases encompass UTILLdb, a repository for pea EMS mutants (Dalmais et al. 2008); PMD, dedicated to pea genetic markers (Kulaeva et al. 2017); and the Pea Genome Database, which includes the pea ‘ZW6’ reference genome (Yang et al. 2022a). In addition, comprehensive sites such as the Pulse Crop Database and the Pulse Crop Breeding and Genetics cater to cool-season legume research (Sanderson et al. 2019; Humann et al. 2019). The SeedStor allows for the search and ordering of pea germplasm resources, and also enables the querying of photos and phenotypic information for different germplasm resources (Horler et al. 2018). These databases collectively offer invaluable resources for advanced pea research (Table 2).

**Table 2:** Pea databases

Database	URL	Description	References
UTILLdb: URGV TILLING pea database	http://urgv.evry.inra.fr/UTILLdb	A database of pea EMS mutants	Dalmais et al. (2008)
Pea Marker Database (PMD)	http://www.peamarker.arriam.ru	A database of pea genetic marker	Kulaeva et al. (2017)
Pea genome database	https://www.peagdb.com	A database of pea ‘ZW6’ reference genome	Yang et al. (2022a)
Pulse crop database	https://www.pulsedb.org	A databsae of cool-season legume genetics, genomics and breeding	Humann et al. (2019)
Pulse crop breeding & genetics	https://knowpulse.usask.ca	A databsae of cool-season legume genetics, genomics and breeding	Sanderson et al. (2019)
SeedStor	https://www.seedstor.ac.uk/	A databsae of searching and ordering pea and other crop germplasm resources	Horler et al. (2018)

## Plant architecture

**Fig. 1:**
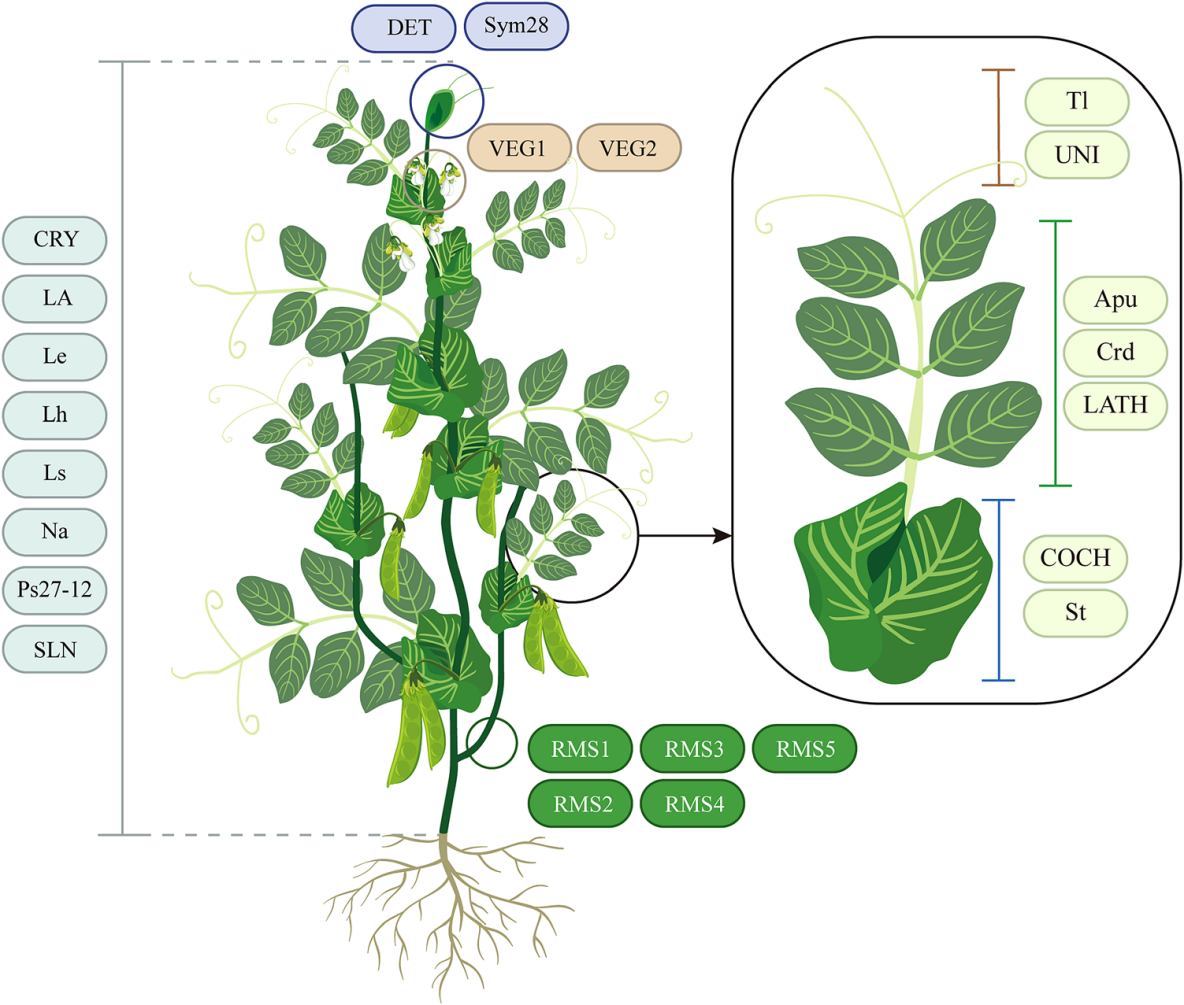
Genes related to plant architecture in pea. *CRY*, *La*, *Le*, *Lh*, *Na*, *Ps27-12* ,and *SLN* regulate plant height. Branch number is controlled by *RMS1*, *RMS2*, *RMS3*, *RMS4*, and *RMS5*. *DET* and *Sym28* participate in the determination of stem growth habit. *VEG1* and *VEG2* regulate inflorescence development. *Tl*, *UNI*, *Apu*, *Crd*, *LATH*, *COCH*, and *St* participate in the leaves development

The architecture of a plant primarily encompasses leaf morphology, stem growth habits, and branching ability among other aspects. Peas, being annual climbing plants, require a trellis for cultivation. Therefore, modulating plant architecture is a pivotal direction in breeding towards simplified cultivation, which on one hand, conserves resources and labor during field management, and on the other hand, enhances pea population yield through the development of a more rational plant structure. In this section, we provide an overview of the functional genes associated with pea plant structure to assist breeders in augmenting pea yield (Table 3, Fig. 1).

**Table 3:** Pea loci/genes regulating plant architecture

Loci/gene	Encoded protein	Mutant phenotypes	References
*Apu*	LOB transcription factor	Lacks the pulvinus	Chen et al. (2012)
*COCH*	BOP-like protein	Stipules degradation	Couzigou et al. (2012)
*Crd*	YUCCA	Reduced leaf vein density	McAdam et al. (2017b)
*CRI*	MYB transcription factor	Leaf polarity defects	Tattersall et al. (2005)
*CRY*	Della	Dwarf	Weston et al. (2008)
*DET*	Terminal flower 1	Apparent terminal flower	Foucher et al. (2003)
*LA*	Della	Dwarf	Weston et al. (2008)
*LATH*	WOX1 transcription factor	Narrow compound leaves	Zhuang et al. (2012)
*Le*	Gibberellin 3-beta-dioxygenase	Dwarf	Lester et al. (1997)
*Lh*	Ent-kaurene oxidase	Dwarf	Davidson et al. (2004)
*Ls*	Copalyl diphosphate synthase	Dwarf	Ait‐Ali et al. (1997)
*Na*	Ent-kaurenoic acid oxidase	Dwarf	Davidson et al. (2003)
*Ps27-12*	Gibberellin 20-oxidase	-	García-Martínez et al. (1997)
*RMS1*	Carotenoid cleavage dioxygenase	Increased branching	Sorefan et al. (2003)
*RMS2*	Auxin receptors	Increased branching	Ligerot et al. (2017)
*RMS3*	Strigolactones receptor	Increased branching	de Saint Germain et al. (2016)
*RMS4*	F-box	Increased branching	Johnson et al. (2006)
*RMS5*	Carotenoid cleavage dioxygenase	Increased branching	Johnson et al. (2006)
*St*	C2H2 zinc finger transcription	Stipules reduced	Moreau et al. (2018)
*SLN*	Gibberellin 2-Oxidase	Dwarf	Martin et al. (1999)
*Sym28*	CLAVATA2	Apical stem fasciation	Krusell et al. (2011)
*Tl*	HD-ZIP transcription factor	Lacking tendrils	Hofer et al. (2009)
*UNI*	LFY/FLO homologue	No rachis or tendrils	Hofer et al. (1997)
*VEG1*	MADS transcription factor	Secondary inflorescences into vegetative branches	Berbel et al. (2012)
*VEG2*	bZIP transcription factor	Tertiary inflorescences	Sussmilch et al. (2015)

### Leaf morphology

The mature wild-type pea leaf exhibits a compound pinnate structure, comprising a basal pair of foliaceous stipules, a pair of proximal leaflets, two pairs of distal tendrils, and a terminal tendril (Gourlay et al. 2000). The pulvinus, a pivotal juncture between the compound leaf and petiole, regulates diurnal leaf movement. The *Apulvinic* (*Apu*) locus is a critical region governing pulvinus formation, encoding a gene orthologous to *MtELP1*, which conservatively regulates pulvinus development in leguminous crops (Chen et al. 2012). Compound leaves contain veins that deliver water and inorganic salts while also facilitating the export of photosynthetic products. The *Crispoid* (*Crd*) locus encodes a YUCCA protein responsible for regulating vein distribution in compound leaves, subsequently influencing photosynthetic efficiency (McAdam et al. 2017b). The *CRISPA* (*CRI*) locus encodes an MYB transcription factor that governs multiple characteristics of pea leaves, encompassing lamina shape, length, position and polarity (Tattersall et al. 2005). The *LATHYROIDES* (*LATH*) locus encodes a WUSCHEL-related homeobox1 (WOX1) transcription factor with a conserved role in dictating organ lateral growth. In *Lath* mutant, both the compound leaves and the stipules are narrowed, and narrower leaflets are observed instead of tendrils (Zhuang et al. 2012). The *UNIFOLIATA* (*UNI*) mutant exhibits rachis or tendrils replaced by a short petiole and pulvinus bearing a single leaflet. Hofer et al. identified this locus as encoding a LFY/FLO homologue protein through forward genetics (Hofer et al. 1997). In stipule development, the *COCHLEATA* (*COCH*) and *Stipules reduced* (*St*) loci play critical roles. They respectively encode a BOP-like protein and a C2H2 zinc finger transcription factor, both of which interact to cooperatively regulate stipule size (Couzigou et al. 2012; Moreau et al. 2018). The *Tl* locus controls pea tendril development by encoding an HD-ZIP transcription factor. In *Tl* mutants, tendrils are replaced by compound leaves (Hofer et al. 2009) (Fig. 1).

### Plant height

An ideal pea plant phenotype should have a shorter stature, promoting upright growth and reducing susceptibility to lodging (Tar’an et al. 2003). Many factors control plant height, with most research focusing on hormonal influences. Several hormones, including gibberellins, auxins (IAA), cytokinins (CTK), brassinosteroids, and ethylene, directly influence plant height. Among these, gibberellins, specifically related to synthesis, degradation, and signal transduction, are the most extensively researched in peas (Kuraishi and Muir 1964). GA_1_, a primary active form of gibberellin in peas, has a synthesis and degradation process heavily influencing plant height (Grindal et al. 1998). Gibberellin metabolism is categorized into three stages (Hedden and Phillips 2000). The initial phase takes place in plastids, where ent-kaurene is derived from trans-geranyl geranyl diphosphate. In this phase, the *Ls* locus encodes Copalyl diphosphate synthase, playing a pivotal role (Ait‐Ali et al. 1997). The subsequent stage reactions transpire outside the plastids, transforming ent-kaurene into GA_53_. In this phase, the *Lh* and *Na* loci encode ent-kaurene oxidase and Ent-kaurenoic acid oxidase, respectively, catalyzing multiple reactions (Davidson et al. 2003, 2004). The final stage of GA_1_ synthesis occurs in the cytoplasm, with GA_53_ being converted to GA intermediates and bioactive GA_1_ by oxidation steps catalyzed by dioxygenases. The *Ps27-12* and the *Le* locus encode GA 20-oxidases and GA 3-oxidases, respectively, culminating in the synthesis of GA_1_ (Lester et al. 1997; García-Martínez et al. 1997). Conversely, the *SLENDER*(*SLN*) locus encodes GA 2-oxidases that deactivate GAs. In the process of GA signal transduction, the *CRY* and *LA* loci each encode DELLA proteins, which act as negative regulators of GA signaling (Weston et al. 2008).

### Branching

For many years, IAA and CTK were believed to be the primary hormones controlling plant branching. However, this perspective shifted when Gomez-Roldan et al. discovered the inhibitory effect of strigolactone (SL) on pea branching, paving the way for research into SL’s role in regulating plant branching (Gomez-Roldan et al. 2008). This discovery in peas was attributed to the fact that many of its branching mutants are associated with SL. The *RAMOSUS 1* (*RMS1*) and *RAMOSUS 5* (*RMS5*) Loci encode two members of the carotenoid cleavage dioxygenase family (PsCCD8 and PsCCD7, respectively), which play crucial roles in SL synthesis (Sorefan et al. 2003; Johnson et al. 2006). These CCDs function downstream of the DWARF27 (D27) isomerase and together catalyze the synthesis of carlactone, a pivotal intermediate in SL biosynthesis. The *RAMOSUS 3* (*RMS3*) and *RAMOSUS 4* (*RMS4*) genes, essential for the SL response, encode the SL receptor (homologous to AtD14 in *Arabidopsis*) and an F-box protein (homologous to AtMAX2 in *Arabidopsis*), respectively (de Saint Germain et al. 2016; Johnson et al. 2006). The *RAMOSUS 2* (*RMS2*) locus encodes an F-box protein from a small family of auxin receptors. It acts as an intermediary in the signal transduction, allowing IAA to promote the synthesis of SL, forming a homeostatic feedback loop (Ligerot et al. 2017). The unique loci, *VEGETATIVE1* (*VEG1*) and *VEGETATIVE2* (*VEG2*), encode the MADS box gene FULc and the bZIP transcription factor FD, respectively (Sussmilch et al. 2015; Berbel et al. 2012). Instead of directly regulating pea branching development, they suppress the transition from vegetative to reproductive growth, thereby leading to increased branching.

### Stem growth habit

The growth habit of the pea stem is a crucial agronomic trait closely linked to the duration of its growth period, yield, and plant height (Foucher et al. 2003). Depending on when apical stem growth terminates, the majority of pea cultivars can be sorted into two main stem architectural types: determinate and indeterminate (Baig et al. 2003). The *DETERMINATE* (*DET)* locus encodes a protein homologous to *Terminal Flower 1* (*TFL1*), which functions to preserve the destiny of the inflorescence meristem in peas. In its mutant, the apical meristem is replaced by a floral structure, leading the pea to transition from unlimited growth to a limited growth form. The *Sym28* locus encodes a CLAVATA2 protein. Through screening of EMS-induced mutants, it was found that when this gene is mutated, shoots in the reproductive phase produce additional flowers, the stem becomes fasciated, and the regular phyllotaxis is disrupted (Krusell et al. 2011).

### Breeding applications of important loci related to plant architecture

Among the various cloned loci that control pea plant architecture, the *Le* locus is perhaps the most renowned for determining internode length and has the widest application in breeding (Mendel 1865). It plays a pivotal role in achieving semi-dwarf breeding in peas. The *Tl* locus, an intriguing one, has been instrumental in the breeding of leafy peas in China. Owing to the replacement of its tough tendrils by compound leaves, superior-tasting varieties like ‘Yunwan No.1’ have been developed. The *DET* locus determines the determinacy of pea stem growth (Foucher et al. 2003). Similarly, other leguminous crops, such as soybeans, have analogous loci, *Dt1* and *Dt2*, which govern stem growth patterns, including determinate, semi-determinate, and indeterminate growth (Ping et al. 2014; Liang et al. 2022; Liu et al. 2010). These loci have been utilized to develop varieties that are dwarfed, resistant to lodging, and mature uniformly (Tian et al. 2010). However, pea varieties developed using the *det* allele, like ‘Determinantnyi VSKhI’, have a lower yield compared to traditional indeterminate varieties, preventing them from becoming the primary loci for breeding modifications (Kondykov et al. 2006; Sinjushin et al. 2022).

As an enhancement to the *DET* locus, certain Russian cultivars have identified and utilized the *Deh* locus, which leads to an early cessation of apical meristem growth. Although little genetic information about this locus is currently available, numerous Russian varieties, including ‘Flagman’, have started utilizing it (Sinjushin et al. 2016). There might exist five loci controlling fasciation: *Fa*, *Fas*, *Fa2*, *Nod4*, and *Sym28* (Marx and Hagedorn 1962; Sidorova and Uzhintseva 1995; Święcicki and Gawlowska 2004; Gawlowska and Swiecicki 2016; Krusell et al. 2011). Early on, British breeders capitalized on these traits to cultivate the ‘Mummy pea’ variety. While such varieties offer consistent maturation and harvesting convenience, their concentrated apical inflorescences make them prone to lodging (Sinjushin 2013). Recently, efforts have been made to utilize the double mutants *det fa*, resulting in plants showcasing an apical raceme, often bearing more than ten flowers on abbreviated pedicels (Kondykov et al. 2006; Zelenov et al. 2012). Given its floral arrangement’s resemblance to lupins, this trait is termed the ‘lupinoid’. Regrettably, to date, there are no registered cultivars with this phenotype. The *AFILA* (*AF*) locus, regarded as a standout in pea breeding, remains uncloned (Demason et al. 2013; Mishra et al. 2009; Gourlay et al. 2000). However, A recent preprint article has offered new speculations (Tayeh et al. 2023). Using *af* mutants, which display a semi-leafless phenotype, the problem of pea lodging can be mitigated, greatly enhancing both yield and quality (Yang et al. 2022b). Presently, semi-leafless pea varieties account for over 95% of the total dry pea production in western Canada and more than 80% in the EU (Acikgoz et al. 2009; Tran et al. 2022). Considering the reduced number of compound leaves in *af* mutants, which might impact photosynthetic efficiency, researchers have innovatively combined *af* with *uni*, developing a phenotype termed ‘chameleon’ (Zadorin et al. 2014; Zelenov et al. 2013). Compared to the single *af* mutant, the *af uni* has a few tendrils replaced by leaves (Marx 1987). Currently, several registered varieties in Russia, such as ‘Spartak’ and ‘Sibirskii’, utilize this phenotype (Zelenov et al. 2013; Sinjushin et al. 2022).

## Symbiotic nitrogen fixation in pea

Similar to other leguminous plants, peas possess the SNF capability. Although peas can fix nitrogen at rates up to 165 kg/ha, the typical fixation range under field conditions lies between 40 and 60 kg/ha (Bourion et al. 2007). This symbiotic relationship facilitates the fixation of atmospheric N_2_. On one hand, it supports the pea’s growth; on the other, it enriches the soil. Unlike industrial nitrogen fixation, SNF does not rely on fossil fuels and is less susceptible to losses through digestion, volatilization, and leaching, making it an ecologically friendly nitrogen source. Over the past two decades, since the first cloning of the SNF-related gene, *NIN* (Schauser et al. 1999), researchers have identified several key genes linked to pea SNF from various germplasm resources. These discoveries have been pivotal in breeding new pea varieties with enhanced nitrogen fixation properties. Here, we provide a comprehensive overview of the genomic research and breeding applications pertaining to pea nodulation during this period (Table 4).

**Table 4:** Pea loci/genes regulating symbiotic nitrogen fixation

Loci/gene	Encoded protein	Mutant phenotypes	References
*Coch*	BOP-like protein	Abnormal nodules	Couzigou et al. (2012)
*Lyk9*	Receptor protein kinase	–	Leppyanen et al. (2017)
*LykX/Sym2*	Receptor protein kinase	Nod^+/–^	Sulima et al. (2017)
*K1*	Receptor protein kinase	Nod^−^/Nod^+/–^	Kirienko et al. (2018)
*Nod3*	Glycosyltransferase	Nod^+/+^	Schnabel et al. (2011)
*Sym7*	GRAS transcription regulator	Nod^−^	Kaló et al. (2005)
*Sym8/Sym20*	Ion channel	Nod^−^	Edwards et al. (2007)
*Sym9/Sym30*	Calcium/calmodulin-dependent protein kinase	Nod^−^	Mitra et al. (2004)
*Sym10*	Receptor protein kinase	Nod^−^	Madsen et al. (2003)
*Sym19/Sym41*	Receptor protein kinase	Nod^−^/Fix^−^	Endre et al. (2002) and Stracke et al. (2002)
*Sym28*	Receptor protein kinase	Nod^+/+^	Krusell et al. (2011)
*Sym29*	Receptor protein kinase	Nod^+/+^	Krusell et al. (2002)
*Sym33/Sym11*	CYCLOPS family	Nod^−^/Fix^−^	Ovchinnikova et al. (2011)
*Sym34*	GRAS transcription regulator	Nod^−^	Shtark et al. (2016)
*Sym35*	Nitrogen netabolism regulator NIN	Nod^−^	Borisov et al. (2003)
*Sym37*	Receptor protein kinase	Nod^+/–^	Zhukov et al. (2008)
*Sym40*	Ethylene response factor	Fix^−^	Nemankin (2011)
*WOX5*	Homeobox transcription factor	–	Osipova et al. (2012)
*KNOX3*	TALE/KNOX homeobox family	–	Azarakhsh et al. (2015)

### Genes cloned for symbiotic nitrogen fixation in peas

The *Sym29* locus was the first to be identified in relation to SNF in peas, and its mutants exhibit both supernodulation and nitrate tolerance (Krusell et al. 2002). *PsSym29* encodes a CLAVATA1-like receptor kinase that is homologous to both *Hypernodulation And Aberrant Root* (*LjHAR1*) and *Super Numeric Nodules* (*MtSUNN*) (Searle et al. 2003; Krusell et al. 2002). Grafting experiments have shown that the supernodulation phenotype observed in mutant *Sym29* is determined by the shoot apex, suggesting its potential involvement in the long-distance regulatory process of nodulation, known as autoregulation of nodulation (AON) (Tsyganov et al. 2013). Another gene that might play a role in the AON process is *Sym28*, which encodes a leucine-rich repeat receptor kinase similar to *AtCLAVATA2*. The *sym28* mutant exhibits both supernodulation and fasciation (Krusell et al. 2011). In addition to the two genes mentioned above, many of the reported genes related to SNF in peas are receptor protein kinases, such as *PsLyk9* (Leppyanen et al. 2017), *PsLykX* (Sulima et al. 2017)*, PsK1* (Kirienko et al. 2018), *PsSym10* (Madsen et al. 2003), *PsSym19* (Stracke et al. 2002; Endre et al. 2002), and *PsSym37* (Zhukov et al. 2008). Among these, *PsSYM10*, *PsSYM37*, and *PsK1* are likely involved in forming complexes for nod factor binding and play significant roles in the initiation of infection and the formation of infection threads.

Transcription factors play a pivotal role in the SNF process of leguminous plants (Griesmann et al. 2018). In the early signaling during nodulation, both *PsSym7* and *PsSym34* are crucial; their mutants fail to form nodules. Both genes encode a GRAS transcription regulator: *PsSym7* is orthologous to *Nodulation Signaling Pathway 2* (*MtNSP2*) (Kaló et al. 2005), while *PsSym34* is orthologous to the *Nodulation Signaling Pathway 1* (*MtNSP1*) gene (Shtark et al. 2016). *PsSym33* corresponds to the *M. truncatula Interacting Protein With Dmi 3* (*MtIPD3*) gene (Ovchinnikova et al. 2011). The *sym33* mutant manifests reduced nodulation or forms non-functional nodules. This is due to the intense defensive response triggered by rhizobial inoculation after the *Sym33* mutation, preventing nodule formation (Tsyganova et al. 2019). Similarly, mutations in *Sym40* locus, which encodes a negative regulator of the cytokinin response transcription factor, inhibit nodule formation because of the elicited intense defensive response (Nemankin 2011; Ivanova et al. 2015). Nodulation signals subsequently target *PsSym35*, a nitrogen metabolism regulator analogous to *Nodule Inception* (*LjNIN*) (Borisov et al. 2003). *PsSym35* enhances the transcription of genes related to nodulation, promoting nodule formation. Through reverse genetics, transcription factors *PsKNOX3* and *PsWOX5* were identified as pivotal to nodule formation (Osipova et al. 2012; Azarakhsh et al. 2015). Furthermore, *coch* is a unique mutant variant, characterized by the typical bifurcation of its nodules and the production of multiple medullary and root structures within its meristematic tissues. Research has shown that *PsCoch* encodes a BOP-like transcription factor, which concurrently regulates the development of multiple organs in pea plants (Ferguson and Reid 2005).

In addition to receptor protein kinases and transcription factors, several other genes play crucial roles in pea SNF. *PsNod3* encodes a glycosyltransferase, mutations in this gene result in the formation of super nodules (Schnabel et al. 2011). *PsSym8* and *PsSym9* encode a potassium ion channel protein and calcium/calmodulin-dependent protein kinase (CCaMK), respectively, both presumed to have a vital role in deciphering nuclear calcium spikes in the nod factor signal transduction pathway (Kaló et al. 2005; Edwards et al. 2007).

### Breeding applications of symbiotic nitrogen fixation in peas

The symbiotic interactions between legume and rhizobial bacteria are estimated to contribute between 91 and 163 million tons of nitrogen annually, with agriculture utilizing 65% of this contribution (Burris and Roberts 1993). Peas primarily satisfy their nitrogen demand for growth and development through SNF, occasionally leaving excess nitrogen in the soil for subsequent crops (Wysokinski and Lozak 2021). Implementing a crop rotation system with peas and cereal or oilseed crops can enhance nitrogen fertilizer use efficiency and overall crop yield (Karkanis et al. 2016; Dowling et al. 2021). The SNF rate in peas is influenced by multiple factors including cultivar characteristics, tillage practices, rotation frequency, inoculant formulation, and soil nitrogen conditions (Dhillon et al. 2022). Among these factors, breeding pea cultivars with high SNF capabilities stands as a direct approach to alter the fixation rate. Genetic variation in the number and weight of nodules in peas has been observed, showing a positive correlation with SNF capability (Abi-Ghanem et al. 2013). Since the 1980s, researchers have identified over 40 *Sym* mutants, several *Nod* mutants, and other genes associated with SNF in peas. While many have been successfully cloned (as shown in Table 4), others have been located on genetic maps (Tsyganov and Tsyganov 2020). Utilizing these mutants, breeders have initiated breeding programs, leading to the development of pea varieties with enhanced nitrogen-fixing capabilities (Dhillon et al. 2022). Sidorova et al. cultivated the ‘Druzhnaya’ variety by amalgamating dominant and recessive alleles from two control super-nodulation loci, *Nod4* and *Nod5*. This variety demonstrated enhanced nitrogen fixation capabilities and yield as compared to its progenitors (Sidorova 2011). Novák et al. (2009) utilized a supernodulating pea mutant *RisfixC*, alongside forage pea cultivars to breed supernodulating forage pea derivatives. Beyond breeding solely for SNF with rhizobia, breeders have also considered the holistic interactions of peas with nodule bacteria, Arbuscular mycorrhiza, and other plant growth-promoting bacteria (Shtark et al. 2012).The effectiveness of interactions with beneficial soil microbes (EIBSM) was assessed, culminating in the development of the inaugural pea cultivar ‘Triumph’. This cultivar, a milestone in the annals of legume breeding, is distinguished for its intentionally enhanced EIBSM (Dhillon et al. 2022).

Numerous attempts have been made in the realm of SNF breeding in peas, yet there remains a wide scope for further efforts. Initially, an abundance of super-nodulating pea mutants such as *sym28*, *sym29*, *nod1*, *nod2*, *nod3*, *nod4*, *nod5*, and *nod6* have been identified (Tsyganov and Tsyganova 2020). However, to date, only a handful of these mutants have been employed in pea nitrogen fixation breeding, leaving many yet to be utilized. Subsequently, prior endeavors to cross super-nodulating pea mutants with conventional pea cultivars to augment nitrogen fixation did not fully succeed due to resultant lower yields, diminished biomass, or stunted growth (Dhillon et al. 2022). This may be attributed to the energy-intensive nature of SNF, which competes with the above-ground parts for carbohydrates (Voisin et al. 2007). Hence, enhancing plant photosynthetic carbon fixation capability while improving SNF becomes crucial. Lastly, since SNF is a subterranean trait, phenotypic acquisition often requires plant destruction, making direct observation challenging in conventional breeding. Therefore, developing corresponding KASP markers based on cloned pea nitrogen fixation genes is of paramount importance (Raina et al. 2023). With the aid of molecular marker-assisted selection breeding, the task of tracking high SNF capacity lines in each generation becomes simpler, significantly accelerating the pea breeding work aimed at SNF. It is anticipated that in the near future, by augmenting the nitrogen fixation capacity of peas, there will be an improvement in both the yield and protein content of the crop. In addition, this advancement is expected to contribute to soil fertility, consequently reducing the necessity for nitrogen fertilizer applications in crops rotated with peas.

## Flowering time

**Fig. 2:**
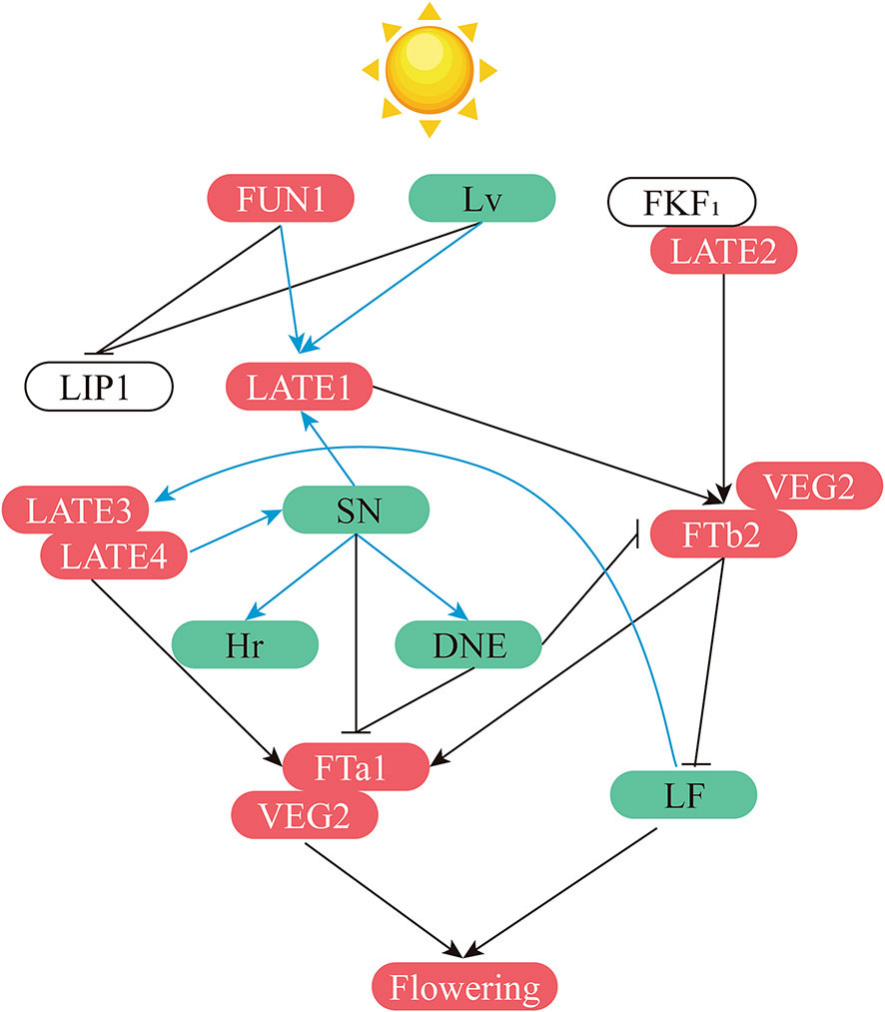
Models delineate the interactions among genes regulating flowering time in pea. Genes promoting flowering are highlighted in red, while those inhibiting are in green. Blue lines represent genetic epistasis between loci, and black lines indicate transcriptional regulation between genes

Legumes can be classified into two distinct clades based on their flowering-time control. Warm season crops, such as soybean and common bean, require short days to flower. Conversely, temperate, cool-season crops like pea, lentil (*Lens culinaris*), and chickpea (*Cicer arietinum*) are long-day plants (Nelson et al. 2010). The ancestral wild species of legumes, due to their varied origins, necessitated strict photoperiodic induction for flowering. However, mutations in many genes controlling photoperiod have occurred over time (Xia et al. 2012; Weller et al. 2012). Through selective breeding, these mutations have enabled present-day legume crops to adapt to varying photoperiods, allowing for cultivation across diverse latitudinal conditions (Dong et al. 2022, 2021; Li et al. 2021; Lu et al. 2020, 2017; Williams et al. 2022). Among these crops, the pea exhibits the broadest distribution, possesses the most varied phenology, and is the most thoroughly understood from a genetic standpoint. As a result, it has become the pioneering model crop for studying photoperiodism in legumes (Weller et al. 1997; Berry and Aitken 1979). In this section, we present a comprehensive overview of the functional genes related to the flowering time of peas. This insight is intended to aid breeders in enhancing the adaptability of the pea crop (Table 5, Fig. 2).

### Photoperiod and flowering time

Plants utilize a range of photoreceptors to sense light, among which cryptochromes and phytochromes are notable (Möglich et al. 2010; Casal 2013). Specifically, Phytochrome A (PhyA) plays an integral role in detecting red and far-red light. In the absence of light, PhyA is dispersed in the cytoplasm. However, after a brief exposure to red or far-red light for approximately five minutes, PhyA is observed to translocate to the nucleus. During this process, a portion of Pr is converted to Pfr, commencing the transmission of light signals within the plant (Casal et al. 2014). The cloning of the pea’s PhyA gene (*FUN1* locus) was significantly advanced due to the identification of a dominant, gain-of-function pea *phyA-3D* mutant, which exhibited amplified PhyA responses (Weller et al. 2004). A mutation in its coding region impedes the light-induced degradation of PhyA, which consequently affects the internal level of active PhyA in peas. This leads mature *phyA-3D* mutant plants to adopt a dwarf phenotype and exhibit early flowering, regardless of the photoperiod. The *Lv* locus encodes Phytochrome B (PhyB) in peas. Mutation *lv* results in early flowering under short-day (SD) conditions (Weller et al. 2001). In other plant species, the nuclear PhyA Pfr negatively regulates several proteins via direct interactions, including the well-studied gene, *Cop1* (Lau and Deng 2012; Ang et al. 1998). While this regulatory mechanism hasn’t been delineated in peas, the *COP1* gene has been successfully cloned from the *Light-Independent Photomorphogenesis1* (*LIP1*) mutant (Sullivan and Gray 2000). Interestingly, the *lip1* mutant not only has a wild-type *COP1* transcript but also an enhanced *COP1* transcript that features an internal in-frame duplication of 894 base pairs. However, the origin of this transcript remains unclear (Sullivan and Gray 2000).

Early genetic research, utilizing controlled SD conditions to investigate the natural variation for flowering time, identified five key loci: *STERILE NODES* (*SN*) on LGVII, *DIE NEUTRALIS* (*DNE*) on LGIII, *LATE FLOWERING* (*LF*) on LGII, *HIGH RESPONSE TO PHOTOPERIOD (HR)* on LGIII, and *PHOTOPERIOD (PPD)* as well as *EARLY* (*E*) on LGVI. With the exception of the last two loci, all have been cloned (Murfet 1971, 1973; Williams et al. 2022). The *sn* mutations promote early flowering, reduce the reproductive phase, and suppress basal branching under SD conditions. Employing classical genetic techniques, The *Sn* was positioned between markers Aldo and Pip2 on LGVII and was ultimately determined to encode an ortholog of LUX (Hazen et al. 2005; Liew et al. 2014). Through phenotypic observations comparing single, double, and triple mutants of *SN*, *HR*, and *DNE*, it was conclusively established that the *SN* locus is epistatic over the *HR* and *DNE* loci (Liew et al. 2014). The *HR* locus encodes a direct homolog of Early Flowering 3 (ELF3). Its mutation induces early flowering under SD conditions, playing a crucial role in the pea’s spread from low to high latitude areas (Weller et al. 2012). The *DNE* locus encodes the ortholog of *Arabidopsis* Early Flowering 4 (ELF4), which has been demonstrated to restrict flowering under non-inductive SD conditions and influence a graft-transmissible flowering signal (Liew et al. 2009). The *LF* locus encodes Terminal Flower 1 (TFL1) homologs and plays a role in prolonging the vegetative phase by delaying floral initiation and the vegetative-to-I1 inflorescence meristem transition (Foucher et al. 2003). In *Arabidopsis thaliana*, the *Flowering Locus T* (*FT*) gene occupies a pivotal position in the genetic hierarchy governing flowering, integrating signals from photoperiod, temperature, vernalization, and light quality (Corbesier et al. 2007). In peas, there are five FT homolog proteins: FTa1, FTa2, FTb1, FTb2, and FTc. Specifically, FTa1 corresponds to the historical *GIGAS* locus in peas, exhibiting upregulated expression in *sn* and *dne* mutants and downregulated expression in *late3* and *late4* mutants (Hecht et al. 2011; Hasan et al. 2020). FTb2 is crucial for inducing flowering under long-day conditions, with *LATE BLOOMER 1* (*LATE1*) and *LATE BLOOMER 2* (*LATE2*) likely promoting its expression (Ridge et al. 2016). Both FTa1 and FTb2 interact with VEG2 to cooperatively regulate pea flowering (Sussmilch et al. 2015).

Hecht et al. conducted a screening of an ethylmethane sulfonate-mutagenized (EMS) M2 population to identify new photoperiod response loci related to late flowering in long days (LD) (Hecht et al. 2007). Their findings revealed multiple phenotypic classes of late-flowering mutants which helped define several genetic loci, Termed *LATE BLOOMER (LATE)* loci. Of these, the *LATE1*, *LATE2*, *LATE3*, and *LATE4* loci have been successfully cloned (Hecht et al. 2007; Ridge et al. 2016; Hasan et al. 2020). Specifically, the *LATE1* locus encodes a GIGANTEA (GI) ortholog, and its mutants flower late under long-day conditions (Hecht et al. 2007). Through crossbreeding the late1 mutant with other early-flowering mutants, researchers found that the *LATE1* and *DNE* loci exhibit a clear interaction. Specifically, *LATE1* is epistatic to *DNE* concerning the overall phenotype under both SD and LD (Liew et al. 2009). Furthermore, the *SN* locus is epistatic to *Late1* in controlling flower initiation, possibly regulating photoperiod-dependent pea flowering by affecting the transcription of *Late1* (Hecht et al. 2007). The *LATE2* locus encodes a Cycling Dof Factor (CDF) ortholog. Functioning downstream of light signaling, *LATE2* can bind and interact with the blue-light photoreceptor FKF1, regulate the main photoperiod-regulated FT gene, FTb2 (Ridge et al. 2016). *LATE BLOOMER 3* (*LATE3*) and *LATE BLOOMER 4* (*LATE4*), orthologs of Cyclin Dependent Kinase 8 (CDK8) and Cyclin C1 (CYCC1) respectively, are integral components of the CDK8 kinase module within the Mediator complex, playing a pivotal role in plant cell cycle regulation. Their interaction may occur at the genetic level with the *SN* locus, potentially modulating the expression of *FTa1* (Hasan et al. 2020).

### Applications of important loci related to flowering time

The domesticated pea, recognized as one of the eight foundational crops, was among the first plants to be domesticated during the Neolithic period (Lev-Yadun et al. 2000; Zohary 1999). Genetic and cytological studies suggest its probable origin from the northern variety (var. *syriacum*) of the wild *P. sativum* ssp. *humile*, a quantitative LD plant. Subsequently, it expanded eastward to the Indian subcontinent and the Himalayan region, and westward to Mediterranean Europe (Williams et al. 2022). The latitudinal spread of the pea was likely driven by selection for decreased photoperiod sensitivity. This allowed for a consistent completion of its life cycle during the shorter summer growing seasons in cool-temperate regions or under the shorter photoperiods of lower latitudes. The main functional variation at the *HR* locus is widespread in pea germplasm worldwide, distinguishing between winter and spring growth patterns (Weller et al. 2012). Natural mutations in the *SN* locus, including a notable 10-bp deletion, likely underlie the distinctive early flowering observed in the renowned pea cultivar ‘Alaska’. Thomas Laxton developed this variety in the United Kingdom, and it was introduced to the United States around 1880. It gained significant popularity there because of its early maturity and adaptability to a broader range of seasons and climates for cultivation (Shoemaker and Delwiche 1934). Although numerous genes related to pea flowering time have been cloned, much remains to be discovered. Historically identified loci, such as *E*, *PPD*, and *AEROMACULATA* (*AERO1*), are yet to be cloned, offering opportunities for further exploration and application (Weller and Orgeta 2015) (Table 5).

**Table 5:** Pea loci/genes regulating flowering time

Loci/gene	Encoded protein	Mutant phenotypes	References
*DNE*	EFL4 ortholog	Early flowering under SD	Liew et al. (2009)
*FUN1*	Phytochrome A	Photoperiod sensing	Weller et al. (2004)
*GIGAS*	FT ortholog	Late flowering under SD	Hecht et al. (2011)
*Hr*	ELF3 ortholog	Early flowering under SD	Weller et al. (2012)
*LATE1*	GIGANTEA ortholog	Late flowering under LD	Hecht et al. (2007)
*LATE2*	Cycling dof factor	Late flowering under LD	Ridge et al. (2016)
*LATE3*	Cyclin dependent kinase	Late flowering under LD	Hasan et al. (2020)
*LATE4*	Cyclin C1	Late flowering under LD	Hasan et al. (2020)
*LF*	Terminal flower1 ortholog	Early flowering under SD	Foucher et al. (2003)
*LIP1*	COP1 ortholog	Light-independent photomorphogenesis	Sullivan and Gray (2000)
*Lv*	Phytochrome B	Photoperiod sensing	Weller et al. (2001)
*SN*	LUX ortholog	Early flowering under SD	Liew et al. (2014)

## Genetic underpinnings of pea floral development

**Fig. 3:**
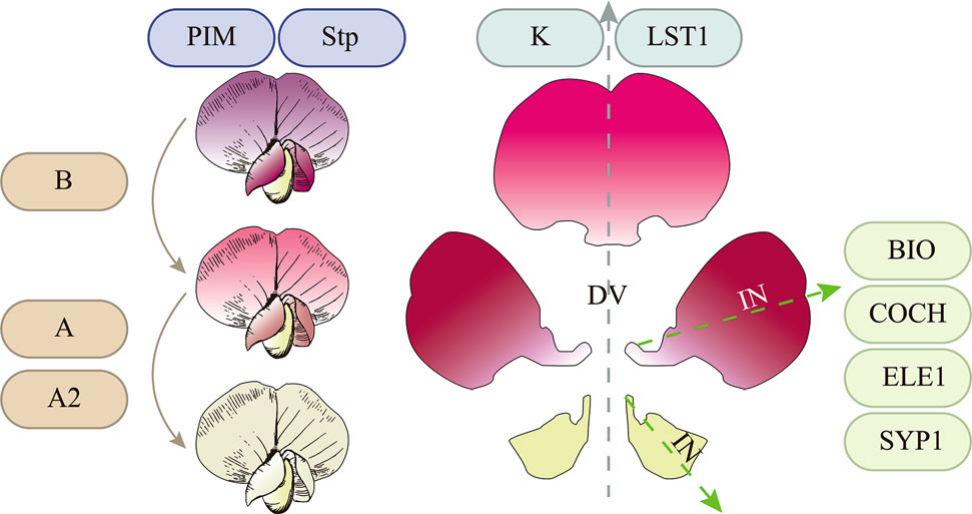
Genes related to flower development in pea. *A*, *A2*, and* B* regulate flower color. *PIM* and *Stp* are involved in petals development. *K* and *LST1* functions to constitute the dorsoventral (DV) asymmetry. *BIO, COCH, ELE1,* and *LST1* functions to constitute the internal (IN) asymmetry

The flower, a reproductive organ in angiosperms, develops through a complex process involving the coordinated action of numerous genes. Peas, as members of the Faboideae subfamily, are characterized by their distinctive papilionaceous flowers comprised of five petals: an upward-facing standard, two lateral wings, and two keels that form a boat-like shape (Yu et al. 2022). Flower development directly impacts a plant’s pollination and fruiting capabilities, ultimately influencing yield (Dohzono and Yokoyama 2010). In this section, we present an overview of the genes associated with pea floral organ development to aid breeders in enhancing pea yield (Table 6, Fig. 3).

**Table 6:** Pea loci/genes regulating flower development

Loci/gene	Encoded protein	Mutant phenotypes	References
*A*	bHLH transcription factor	Lacking anthocyanin	Hellens et al. (2010)
*A2*	WD40	Lacking anthocyanin	Hellens et al. (2010)
*B*	F3′5’H	Pink flower	Moreau et al. (2012)
*Bio*	KIX domain protein	Symmetrical lateral and ventral petals	Li et al. (2019)
*Coch*	BOP-like protein	Two standards and chimeric stamen-wing petals	Couzigou et al. (2012)
*Ele1*	TIFY family transcription factors	Symmetrical lateral and ventral petals	Li et al. (2019)
*K*	TCP transcription factors	Wing petals keel like	Wang et al. (2008)
*Lst*	TCP transcription factors	Abnormal shape in the dorsal petals	Wang et al. (2008)
*Stp*	UFO-like- protein	Flowers only contain sepals and carpels	Taylor et al. (2001)
*Syp1*	ALOG domain protein	Symmetrical lateral and ventral petals	He et al. (2020)
*Pim*	AP1-like transcription factor	Flower within flower	Taylor et al. (2002)

### Floral morphology

In Papilionoideae legumes, zygomorphic flowers are characterized by a distinct corolla with three petal types, displaying both dorsoventral (DV) and internal (IN) asymmetry. As a result, the symmetry of pea flowers has become a focal point in the study of pea floral organ development (Yu et al. 2022). The *K* locus and *LOBED STANDARD 1* (*LST1*) locus encode CYC-like TCP proteins, which act as DV regulators, controlling lateral and dorsal identities, respectively. They are believed to have arisen from the duplication of an ancestral *TCP* gene during the speciation of papilionoid legumes (Wang et al. 2008). In contrast, the *SYMMETRICAL PETAL 1* (*SYP1*) locus encodes an ALOG Domain Protein and functions independently to regulate the IN asymmetry of the petal (He et al. 2020). The *BIGGER ORGANS* (*BIO*) locus encodes a KIX domain protein, while the *ELE1* encodes a member of the TIFY family of transcription factors. These proteins can interact with each other and regulate not only the IN asymmetry of petals but also the overall size of the organ (Li et al. 2019).

The *COCH* locus plays multiple roles and encodes a BOP-like protein. It not only modulates the morphology of root nodules and stipules but also alters floral morphology. While normal flowers have one standard petal, the *coch* mutant manifests with two standard petals and chimeric stamen-wing petals (Couzigou et al. 2012). The *PROLIFERATING INFLORESCENCE MERISTEM* (*PIM*) locus encodes an AP1-like transcription factor. Mutations in *pim* lead to delayed floral meristem specification and abnormalities in the first and second whorl of floral organs (Taylor et al. 2002). *The Stamina Pistilloida (Stp)* locus, encoding a UFO-like protein, is vital for the normal development of flowers, inflorescences, and leaves (Taylor et al. 2001). The *stp* mutant predominantly produces flowers with sepals and carpels.

### Anthocyanidin

Among the pea genes determining flower color, the most notable is the *A* locus, famously utilized in Mendel’s hybridization experiments (Mendel 1865). This locus encodes a bHLH transcription factor, which is extensively distributed in natural populations and plays a pivotal role in determining whether pea flowers are colored or colorless (Hellens et al. 2010). Concurrently identified with the *A* locus was the *A2* locus, which encodes a WD40 protein (Hellens et al. 2010). Together, they are potentially integral components of the MYB–bHLH–WD40 protein (MBW) complex in peas, responsible for regulating anthocyanin-associated gene transcription (Li 2014). The *B* locus encodes a flavonoid 3′,5′-hydroxylase (F3′5′H). The *b* mutants lack glycosylated delphinidin and petunidin, which are the predominant pigments in the purple-flowered wild-type pea, resulting in pink-colored flowers (Moreau et al. 2012).

### Applications of important loci related to floral development

Many of the cloned genes related to floral development exhibit detrimental effects on plant growth and development, making them more suitable for foundational research rather than practical application (Couzigou et al. 2012; Taylor et al. 2001). However, the *BIO* and *ELE1* loci appear to be exceptions, as mutations in these loci result in enlarged organs (Li et al. 2019). Experimental techniques such as VIGS have confirmed that silencing *BIO* or *ELE1* leads to larger pea pods. Consequently, these loci could be vital considerations for future high-yield pea breeding. Inaddition, some uncloned loci play crucial roles in breeding. Among them, *FLOWER NUMBE*R (*Fn*) and *FLOWER NUMBER A* (*Fna*) are paramount (Singer et al. 1999; Sinjushin and Liberson 2016). These loci regulate the number of flowers on a single pedicel. While typical cultivars usually bear two flowers per pedicel, plants with the *Fn Fna* genotype often produce three or even more flowers, undoubtedly contributing to a significant increase in pea yield (Devi et al. 2021, 2018).

While the anthocyanin content in floral organs might not have significant practical implications, as crucial switches in anthocyanin synthesis, they exert dominant effects on other traits requiring anthocyanin. For instance, the *Pu* and *Pur* loci are key determinants of pea’s purple pods (Donkin et al. 1993), and the *D* locus regulates anthocyanin in stipules (Hagh and Azimi 2003; Ellis and Poyser 2002). The functioning of these loci is contingent upon the intact functionality of the *A* locus (Hellens et al. 2010). Thus, ensuring the normal function of anthocyanin synthesis-related loci is paramount when selecting genes for breeding these traits.

## Seeds and pods

**Fig. 4:**
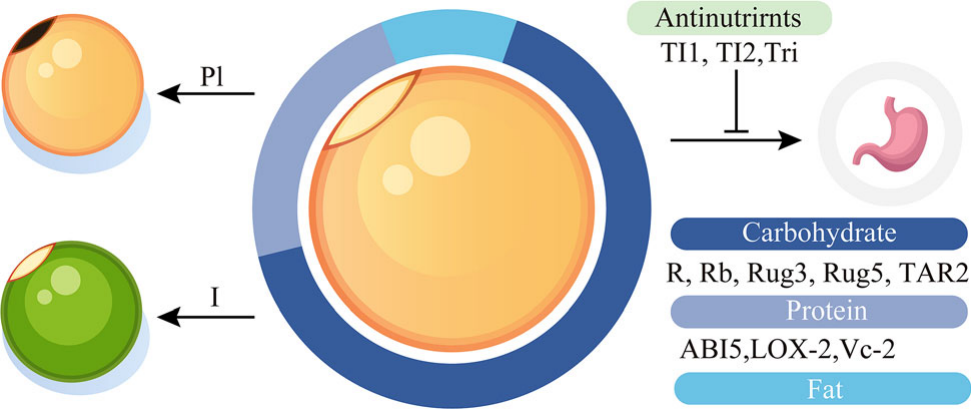
Genes related to seed in pea. *R*, *Rb*, *Rug3*, *Rug5*, and *TAR2* regulate starch synthesis within carbohydrates. *ABI5*, *LOX 2*, and *Vc-2* participate in the protein synthesis. Antinutrients are controlled by *TI1*, *TI2*, and *Tri*. *Pl* has been identified as a key regulator of hilum color. *I* alter the color of cotyledons

The seeds and pods, as the edible parts of the pea plant, play a pivotal role in determining pea yield. Extensive research has been conducted on the development of pea seeds and pods, with particular emphasis on the nutritional quality of the seeds. This section reviews the studies related to genes associated with pea seeds and pods and provides an overview of significant loci utilized in breeding (Table 7, Fig. 4).

**Table 7:** Pea loci/genes regulating seed development

Loci/gene	Encoded protein	Mutant phenotypes	References
*Abi5*	bZIP family transcription factor	Reduced vicilin	Le Signor et al. (2017)
*I*	Stay-Green protein	Green cotyledons	Armstead et al. (2007)
*Lox-2*	Lipoxygenase-2	Less lipoxygenase	Forster et al. (1999)
*Pl*	Polyphenol oxidase	Less hilum pigmentation	Balarynová et al. (2022)
*R*	Starch branching enzyme I	Wrinkled and amylose-rich	Bhattacharyya et al. (1990)
*Rb*	ADP-glucose pyrophosphorylase	Wrinkled and starch-low	Hylton and Smith (1992)
*Rug3*	Plastidial phosphoglucomutase	Almost starchless	Harrison et al. (2000)
*Rug5*	Starch synthase II	Abnormal starch granule and amylopectin	Craig et al. (1998)
*Tar2*	Aminotransferase	Small seeds with reduced starch content	McAdam et al. (2017a)
*Ti1*	Trypsin inhibitor	Low seed protease inhibitory activity	Clemente et al. (2015)
*Ti2*	Trypsin inhibitor	Low seed protease inhibitory activity	Clemente et al. (2015)
*Tri*	Trypsin inhibitor	Low seed protease inhibitory activity	Page et al. (2002)
*Vc-2*	Vicilin polypeptide	Reduced vicilin	Chinoy et al. (2011)

### Genes cloned for seeds in peas

More than half of the nutritional content in pea seeds comprises carbohydrates, predominantly stored as starch, which accounts for approximately 45–50% of the pea seed’s dry weight (Bhattacharyya et al. 1990). Consequently, among all nutritional components, genes associated with starch synthesis are most abundant. Historically, the most notable gene related to starch synthesis is the *Rugosus* (*R*) locus, which was utilized by Mendel during his hybridization experiments. This gene encodes a starch branching enzyme (Bhattacharyya et al. 1990). Its mutation leads to an increase in resistant starch, resulting in the wrinkled-seeded phenotype. The *Rugosus b* (*Rb*) locus encodes ADP-glucose pyrophosphorylase, a crucial enzyme in the starch synthesis pathway. Mutations at this locus reduce starch content by about 50% (Hylton and Smith 1992). Mutations in other genes within the pea starch synthesis pathway also result in anomalies in starch production. For instance, the *Rugosus 3* (*Rug3*) gene encodes plastidial phosphoglucomutase; mutations at this locus yield peas with virtually no starch (Harrison et al. 2000). Meanwhile, the *Rugosus 5* (*Rug5*) gene encodes starch synthase II, and its mutations alter starch granule morphology and the structure of amylopectin (Craig et al. 1998). The *TRYPTOPHAN AMINOTRANSFERASE RELATED 2* (*TAR2*) locus encodes an aminotransferase involved in the auxin biosynthesis pathway. By regulating auxin levels, it subsequently controls both pea seed size and starch content (McAdam et al. 2017a).

In leguminous crops, such as peas, one of the primary distinctions from cereals is the protein content in the seeds. Leguminous seeds contain notably higher protein levels than cereals (Maphosa and Jideani 2017). The primary storage proteins in pea seeds are the globulins, legumin and vicilin. The biosynthesis pathway for these proteins involves more than forty genes (Robinson and Domoney 2021). The previously mentioned *R* and *Rb* loci impact the content of legumin, with mutations leading to a significant reduction in legumin levels (Casey et al. 2001). The *Vc-2* locus encodes a protein associated with vicilin polypeptides (Chinoy et al. 2011), and the *ABA-Insensitive 5* (*ABI5*) locus encodes a bZIP family transcription factor, which is a component of the abscisic acid (ABA) signaling pathway in seeds (Le Signor et al. 2017). Both play crucial roles in vicilin synthesis, and mutations result in a significant reduction of vicilin in the seed. In addition, Lipoxygenases (LOX) are prevalent seed proteins that catalyze the synthesis of hydroperoxides from fatty acids. In peas, the *Lipoxygenases 2* (*LOX2*) locus governs this trait. Through screening of plant resources, natural *lox2* mutant variants were identified, with promoter mutations leading to altered expression (Forster et al. 1999).

Antinutrients are compounds, either natural or synthetic, predominantly found in foods such as grains, beans, legumes, and nuts. These compounds hinder the absorption of vitamins, minerals, and other nutrients (Popova and Mihaylova 2019). Among the antinutrients present in peas are seed protease inhibitors, which can diminish the nutritional quality of pea seeds, impacting various applications in the food and feed industries (Clemente et al. 2015). The *TI1*, *TI2,* and *Tri* locus encode three distinct trypsin inhibitors. Mutations in these loci reduce trypsin inhibitor activity in pea seeds, thereby enhancing their nutritional quality (Clemente et al. 2015; Page et al. 2002).

The *I* locus, responsible for seed color among Mendel’s seven major traits, was cloned early on. It encodes a Stay-Green protein that alters seed color by directing chlorophyll into the chlorophyll degradation pathway (Armstead et al. 2007; Sato et al. 2007). Hilum color, considered one of the domestication traits, is controlled by the *Pl* locus. This locus encodes a Polyphenol oxidase, influencing the oxidation and polymerization of gallocatechin in the seed coat, subsequently leading to hilum pigmentation (Balarynová et al. 2022) (Table 7).

### Applications of important loci related to seeds and pods

Genetic mutations affecting starch synthesis typically result in reduced starch production, leading seeds to accumulate higher sugar levels. Consequently, this elevates the fresh consumption quality of the seeds. Among genes related to starch synthesis, the *R* locus, especially, has been recognized as pivotal in distinguishing dry (*R*) from vegetable cultivars of pea (*r*) (Sinjushin et al. 2022). Recent studies indicate that pea varieties possessing the r allele, in contrast to those with the R allele, have a lower glycemic index. This characteristic aids in preventing postprandial glucose spikes, making it a promising direction for future health-focused breeding initiatives (Petropoulou et al. 2020). While the effects of the *R* locus (wrinkling of seeds) are easily observable, other loci governing seed nutrition, such as the Low-phytate (*Lpa*) locus for phytic acid synthesis (Shunmugam et al. 2015), the *VicB* locus for vicilin control (Lycett et al. 1983), and the *Pea Albumin 1* (*PA1*) and *Pea Albumin 2* (*PA2*) loci for albumin regulation (Eyraud et al. 2013; Vigeolas et al. 2008), have received foundational research attention but prove challenging for current breeding applications. Recently, Zhou et al. utilized the recombinant inbred line population PR-25 to identify several QTLs associated with amino acid concentration and in vitro protein digestibility in peas (Zhou et al. 2023). In contrast, traits affecting the morphology of pea seeds, which are readily visible, are more easily harnessed in breeding. The *Development Funiculus* (*Def*) locus governs the formation of the boundary between the funiculus and seed hilum (Ayeh et al. 2009). Mutants at the *Def* locus lack this boundary, causing the pod to burst open, and their seeds remain firmly attached to the pod, significantly reducing harvest losses. Russian pea breeders identified and exploited this trait early on, and it is noted that almost half of all contemporary Russian pea cultivars possess non-abscising (*def*) seeds (Zelenov 2013).

Beyond the *BIO* and *ELE1* loci, which influence pod size, most genes associated with pod development remain uncloned. Nevertheless, many of them possess significant breeding potential and some have already been utilized in breeding programs. The *N* locus determines pod thickness; its mutation leads to the thicker, crunchy textured pods characteristic of the sugar snap pea type (Wehner and Gritton 1981). The *P* and *V* loci regulate the development of the sclerenchyma of the inner pod, with mutations resulting in the cultivation of the more tender snow pea (Karaca 2019). The *Sin* and *Sin-2* loci control the formation of the pod cord, located at the pod sutures (Ma et al. 2016; McGee and Baggett 1992). By combining traits from the *p*, *v*, *n*, and *sin-2* loci, breeders have developed snap pea varieties with pods that are edible even when fully inflated (Murfet and Reit 1993). The *Dpo* locus influences the dehiscence of pea pods. The *Dpo* allele is predominantly found in wild varieties with dehiscent pods, while the *dpo* allele is mainly present in modern cultivars with indehiscent pods (Weeden 2007). In terms of yield, the *Te*, *Teu*, *Lt*, and *Laf* loci govern pod width, while the *Cotr* and *Curt* loci dictate pod length. These loci are prioritized in the future improvement of edible-podded peas, such as snow peas and snap peas (Ellis et al. 2021). Klein et al. conducted a meta-analysis of quantitative trait loci (QTL) to collate and analyze all yield-related QTLs identified in recent years (Klein et al. 2020). This analysis resolved these QTLs into 27 distinct metaQTLs, several of which exhibited narrow confidence intervals under 2 centiMorgans (cM), encompassing fewer than one hundred underlying candidate genes.

## Resistance genes

Like many crops, peas face a range of abiotic and biotic stresses that can impede their growth, yield, and quality. Ongoing research focuses on understanding resistance to these stresses, aiming to ensure consistent pea production despite varying environmental conditions. Breeding varieties resistant to both biotic and abiotic stressors is an effective strategy for enhancing the productivity of crops, including peas. Thus, understanding the genes related to pea stress resistance and identifying key resistance loci is crucial. This section summarizes the cloned stress-resistance genes in peas and highlights important resistance loci that are yet to be cloned, serving as a reference for future stress-resistant breeding (Table 8).

**Table 8:** Pea loci/genes conferring resistance to stresses

Loci/gene	Encoded protein	Mutant phenotypes	References
*Er1*	MLO protein	High resistance to *Erysiphe polygoni*	Humphry et al. (2011)
*Sbm1*	Eukaryotic translation initiation factor	High resistance to seed-borne mosaic virus	Gao et al. (2004)

### Genes cloned for resistance in peas

Pea productivity is significantly affected by a range of fungal pathogens, with powdery mildew, caused by *Erysiphe* species, being the most detrimental. The *Er1* locus plays a pivotal role in conferring resistance to powdery mildew in peas, encoding the mildew resistance locus O (MLO) protein (Humphry et al. 2011; Fondevilla et al. 2006). The *er1* allele has been identified to grant resistance by obstructing the invasion of *Erysiphe pisi* (*E. pisi*) into pea epidermal cells. In the majority of pea accessions containing the *er1* allele, a vast number of *E. pisi* conidia germinate and develop appressoria. However, these show limited pathogen growth and lack secondary hyphae formation (Iglesias-García et al. 2015). The *Sbm1* locus determines pea’s resistance to the seedborne mosaic virus. The *sbm1* allele represents a non-functional variant of a crucial factor for host susceptibility to the pea seed-borne mosaic virus (PSbMV). This allele inhibits the virus’s genome expression, multiplication, and intercellular movement (Gao et al. 2004).

### Applications of important loci related to resistance

Among the various diseases affecting peas, powdery mildew remains the most prevalent and detrimental. To date, only three genes conferring resistance to *E. pisi* have been described: *er1*, *er2*, and *Er3*. The *Er1* locus, which has been cloned, was initially identified in the local variety ‘Huancabamba’ and is now widely utilized in pea breeding (Iglesias-García et al. 2015). Resistance governed by the *Er2* and *Er3* loci is primarily characterized by a post-penetration hypersensitive response that halts colony growth. While these two loci have not yet been cloned, linked DNA markers are available, enabling marker-assisted breeding (Ghafoor and McPhee 2012). Beyond powdery mildew resistance loci, numerous loci governing other resistances have been discovered. For instance, the *Ruf* locus controls rust resistance (Vijayalakshmi et al. 2005), *Rpv* locus dictates resistance to *Peronospora pisi* (Wingerter et al. 2021), *Rap-2* locus manages Ascochyta resistance (Dirlewanger et al. 1994), *Mo* locus determines mosaic virus resistance (Dirlewanger et al. 1994), *Lr* locus governs bean leaf rool virus resistance (Swiecicki and Timmerman-Vaughan 2005), *Fw-1* and *Fnw* locus controls *Fusarium oxysporum* resistance, and *En* loci control resistances to enation mosaic virus (Mc Phee et al. 2012). Although the genes for these loci have not been cloned, their mutant variants can be employed in hybrid breeding to cultivate more resistant varieties.

## Conclusion and future perspective

Meeting the demands of a growing global population by enhancing yield is a pressing challenge in pea breeding. The surge in genomic data for peas in recent years lays a robust foundation for both fundamental research and innovative breeding strategies. Despite significant efforts devoted to pea breeding over the past years, its yield remains relatively low compared to other leguminous crops. This discrepancy may primarily arise from the focus of breeding objectives in various countries being concentrated on a limited number of traits, such as tendril formation (*Af* locus), dwarf stature (*Le* locus), and powdery mildew resistance (*Er1* Locus), leading to a reduced genetic base. Herein, we discuss several potential strategies for increasing yield and enhancing breeding, as well as how functional genomics can facilitate these processes.

### Utilizing wild resources to enhance resistance breeding

Wild relatives of crops are considered valuable resources for genetic improvement, enabling enhanced adaptability to adverse environmental conditions. During its domestication, pea has experienced several genetic bottlenecks, notably in recent decades of breeding, which have substantially reduced its genetic diversity. However, wild pea species hold immense potential as donors for various essential agronomic traits. *Pisum fulvum* possesses resistance to the pea weevil (Byrne et al. 2008), rust (Barilli et al. 2010), and powdery mildew (Fondevilla et al. 2007). *Pisum elatius* exhibits resistance to *Orobanche crenata* (Valderrama et al. 2004), nematode *Heterodera goettigniana* (Valderrama et al. 2004), PSbMV (Konečná et al. 2014). Therefore, exploiting wild germplasm to identify resistance genes and reintroducing these genes into cultivated pea varieties is likely the most viable approach to achieve sustainable pea production. Enhancing pea’s resistance to pests and diseases can significantly reduce chemical and labor inputs, simultaneously increasing pea yield and quality while mitigating the environmental impact of pesticides.

### Precise breeding through genome editing

At the current stage, pea breeding primarily relies on traditional methods. However, these methods are characterized by lengthy breeding cycles and often excessively depend on the breeders’ experience. (Rubiales et al. 2019). Gene editing technology offers precise genome modifications without the introduction of foreign DNA, holding significant potential for crop improvement. Compared to other crops, the breeding of peas through gene editing is still in its nascent stage. This is primarily due to challenges in its genetic transformation and a scarcity of suitable gene-editing tools. Public acceptance of gene-edited foods might also be a significant factor hindering its progress. However, with recent advancements in pea genetic transformation techniques and the development of appropriate gene-editing tools for peas, breakthroughs are becoming achievable (Li et al. 2023). Bhowmik et al. utilized gene editing in peas to modify lipoxygenase enzymes, swiftly enhancing the aroma and fatty acid profiles of pea seeds from an elite Canadian variety (Bhowmik et al. 2023). This research provides pivotal direction for the future development of precise breeding through genome editing in peas.

### Developing a rational farming system utilizing the nitrogen-fixing ability of peas

Intercropping is a potentially effective yet underexploited strategy that can enhance soil fertility, boost crop yields, minimize environmental damage, and increase farmers’ income (Hauggaard-Nielsen et al. 2009). The combination of nitrogen-fixing legumes with cereals offers an excellent means to improve soil conditions and reduce fertilizer usage. Maize-soybean intercropping, due to its capacity to sustain maize yields while yielding an additional soybean crop within a season, has been widely adopted worldwide, serving as a model for novel pea cultivation methods (Du et al. 2023; Raza et al. 2022). Presently, researchers have embarked on new intercropping practices involving pea-spring wheat (Mamine and Fares 2020), pea-spring maize (Yang et al. 2023), pea-barley (Hauggaard-Nielsen et al. 2001), and pea-oats (Carr et al. 1998), yielding favorable results. Nonetheless, pea intercropping faces multiple challenges, such as optimal intercropped strip allocation, selection of the best intercropping species, and the development of specialized machinery for intercropping sowing and harvesting. These issues necessitate further scientific investigation.

Incorporating leguminous crops into crop rotation systems often leads to higher seed yields in subsequent cereal crops. The increase in soil nitrogen availability observed in the pea-wheat rotation, as evidenced by the A-value, accounts for 8–9% of the seed yield improvement due to rotation effects (N benefit) (Stevenson and Kessel 1996a, b). In Southwest China, autumn-sown peas harvested in spring enhance winter land utilization, increase soil nitrogen levels, and consequently boost farmers’ incomes.

## Data Availability

Data will be made available on request.
